# Clinical Correlates of Autosomal Chromosomal Abnormalities in an Electronic Medical Record–Linked Genome-Wide Association Study: A Case Series

**DOI:** 10.1177/2324709613508932

**Published:** 2013-10-18

**Authors:** Hayan Jouni, Khader Shameer, Yan W. Asmann, Ribhi Hazin, Mariza de Andrade, Iftikhar J. Kullo

**Affiliations:** 1 Division of Cardiovascular Diseases, Mayo Clinic, Rochester, MN; 2 Division of Biomedical Statistics and Informatics, Mayo Clinic, Rochester, MN; 3 Department of Internal Medicine, Wayne State University, Detroit, MI

**Keywords:** copy number variation, genome-wide association studies, loss of heterozygosity, mosaic abnormalities, mosaic deletion, myeloproliferative disorders, prostate cancer, unipaternal disomy

## Abstract

Although mosaic autosomal chromosomal abnormalities are being increasingly detected as part of high-density genotyping studies, the clinical correlates are unclear. From an electronic medical record (EMR)–based genome-wide association study (GWAS) of peripheral arterial disease, log-R-ratio and B-allele-frequency data were used to identify mosaic autosomal chromosomal abnormalities including copy number variation and loss of heterozygosity. The EMRs of patients with chromosomal abnormalities and those without chromosomal abnormalities were reviewed to compare clinical characteristics. Among 3336 study participants, 0.75% (n = 25, mean age = 74.8 ± 10.7 years, 64% men) had abnormal intensity plots indicative of autosomal chromosomal abnormalities. A hematologic malignancy was present in 8 patients (32%), of whom 4 also had a solid organ malignancy while 2 patients had a solid organ malignancy only. In 50 age- and sex-matched participants without chromosomal abnormalities, there was a lower rate of hematologic malignancies (2% vs 32%, *P* < .001) but not solid organ malignancies (20% vs 24%, *P* = .69). We also report the clinical characteristics of each patient with the observed chromosomal abnormalities. Interestingly, among 5 patients with 20q deletions, 4 had a myeloproliferative disorder while all 3 men in this group had prostate cancer. In summary, in a GWAS of 3336 adults, 0.75% had autosomal chromosomal abnormalities and nearly a third of them had hematologic malignancies. A potential novel association between 20q deletions, myeloproliferative disorders, and prostate cancer was also noted.

## Introduction

Genome-wide association studies (GWAS) are identifying novel genomic loci associated with various diseases and quantitative traits.^1,2^ Chromosomal abnormalities such as mosaic deletions, amplifications, and unipaternal disomies are incidentally found on signal intensity analyses in such studies. The clinical correlates of these abnormalities remain poorly defined.^3^ In 2007, the National Human Genome Research Institute funded the electronic MEdical Record and GEnomics (eMERGE) consortium to study and evaluate the utility of high throughput electronic medical record (EMR)–based phenotyping methods to facilitate genomic studies.^4,5^ This approach was successfully applied to GWAS of several quantitative traits including red blood cell indices, erythrocyte sedimentation rate, white blood cells, and PR interval of the electrocardiogram.^4,6-9^

The availability of high-density genotyping data linked to the EMR in the eMERGE consortium offers an opportunity to study the clinical correlates of incidentally found chromosomal abnormalities. The GWAS at Mayo Clinic was conducted to identify loci associated with peripheral arterial disease (PAD). An important area of investigation involves return of incidentally found genetic abnormalities. As a step in this direction, we conducted a detailed EMR review of patients with incidentally found autosomal chromosomal abnormalities and controls without such abnormalities, to ascertain the clinical correlates of these abnormalities.

## Materials and Methods

The study protocol was approved by the Mayo Clinic Institutional Review Board and included 3336 participants: 1687 PAD cases (mean age = 65.8 ± 10.7 years, 64.3% men) and 1649 control subjects (mean age = 60.6 ± 7.3 years, 59.8% men). Written informed consent was obtained. Details of patient recruitment and characteristics have been previously described.^4,6^ Genotyping was performed at the Center of Genotyping and Analysis at the Broad Institute, Cambridge, Massachusetts, using the Illumina Human 660W-Quad V1 genotyping platform that consists of 561 490 single nucleotide polymorphisms (SNPs) and 95 876 intensity-only probes.

**Figure 1.**
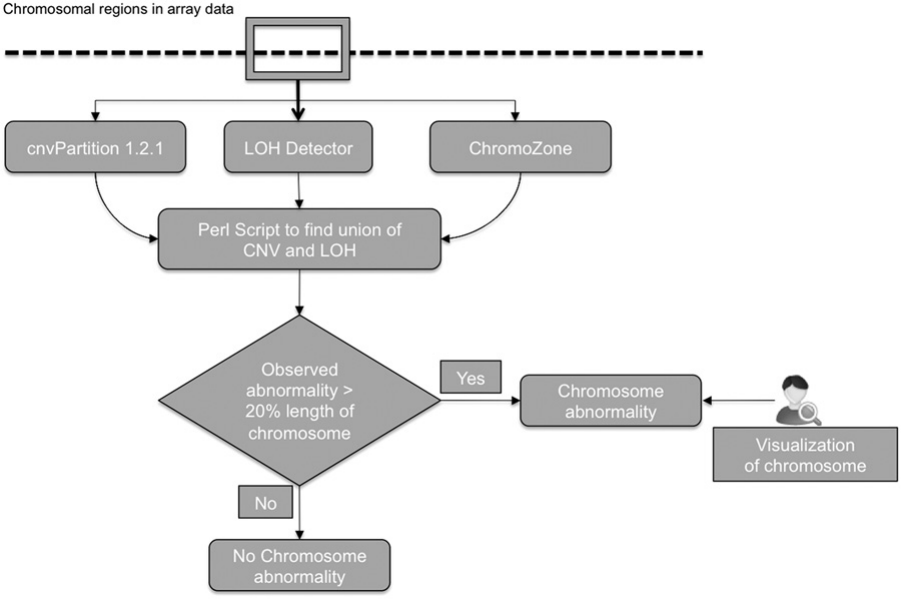
Flowchart of the computational genomics pipeline used to identify chromosomal abnormalities. Three GenomeStudio plug-ins (cnvPartition 1.2.1, LOH detector, and ChromoZone; Illumina, San Diego, CA) were combined and run using log-R ratio (LRR) and B-allele frequency (BAF) data to identify copy number variation (CNV) and loss of heterozygosity (LOH) regions (both copy neutral LOH and heterozygous deletion LOH). The unions of LOH and CNV from these 3 algorithms were combined using a Perl script developed in-house. A chromosomal abnormality was considered to be present when the combined abnormalities exceeded 20% of the chromosome’s total length. The signal intensity data from each abnormal chromosome were visually examined to validate the call.

A genomic data analysis pipe-line that combined 3 GenomeStudio plug-ins (cnvPartition 1.2.1, LOH detector, and ChromoZone; Illumina, San Diego, CA) waas run using log-R ratio (LRR) and B-allele frequency (BAF) data to identify copy number variation (CNV) and loss of heterozygosity (LOH) regions (both copy neutral LOH and heterozygous deletion LOH). CnvPartition 1.2.1 was used to estimate copy number and for annotation of chromosomal regions with CNV. LOH detector was used to detect extended tracts of homozygosity and ChromoZone was used to auto-bookmark for single-sample analysis. The computational genomics pipeline used to define a chromosome abnormality is summarized in Figure 1, and the computational script can be found in the supplementary material (available online at http://HIC.sagepub.com/supplemental). The unions of LOH and CNV from these 3 algorithms were combined using a Perl script developed in-house. A chromosomal abnormality was considered to be present when the combined abnormalities exceeded 20% of the chromosome’s total length. The signal intensity data from each abnormal chromosome were visually examined to validate the call. We used previously established definitions^10^ in classifying the observed chromosomal abnormalities. Deviations from the expected normal bipaternal disomic state were assessed using LRR and BAF data. In a normal study participant, BAF at any locus is expected to be either 0 (AA), 0.5 (AB), or 1 (BB) corresponding to an LRR of 0. Negative deviations of LRR corresponded to deletions whereas positive deviations were compatible with amplifications. Samples with BAF asymmetry and LRR of 0 were classified as unipaternal disomies (UPD).

The EMR of 25 patients with chromosomal abnormalities and 50 randomly selected age-, sex-, and PAD case-matched participants without chromosomal abnormalities (in a 1:2 ratio) were reviewed in detail by one of the authors (HJ) to ascertain associated disease states and other clinical characteristics. The review included all physician notes, radiology studies, laboratory results, and pathology reports.

Five patients had chromosome 20q deletions (Ch20q del), and we identified the common deleted region in these patients. Using the break points for each of the 5 patients, the deleted segments of Ch20q were mapped to the human reference sequence (NCBI36/hg18) in UCSC Genome browser using custom tracks. The genes encoded in the common deleted region were identified from NCBI36/Ensembl54 database using the Bioconductor package bioMart.^11^

## Results

Of the 3336 genotyped study participants, 25 patients (PAD cases: n = 20, controls: n = 5, mean age = 74.8 ± 10.7 years, 64% male, 0.75% of total study participants) were found to have abnormal intensity plots consistent with the following chromosomal abnormalities: mosaic deletions (DEL = 7), mosaic and typical UPD (UPD = 18), and one chromosome with both amplification/UPD. One patient had 2 abnormalities: UPD and DEL. Table 1 lists the type of the chromosomal abnormality as well as the accompanying significant medical conditions ascertained by detailed review of the EMR. Supplementary material includes intensity plots of all patients in this report.

**Table 1.** Patient Characteristics.

Ch	Abnormality	Age	Sex	Atherosclerotic Disease	Hematologic Disorder	Other Significant Medical History
2	UPD	69	Male	None	—	Hemorrhagic stroke due to amyloid angiopathy
3	UPD	77	Male	CAD	—	—
3	UPD	78	Female	CAD/PAD	Vitamin B_12_ deficiency	Sarcoidosis-related myopathy and neuropathy
4	UPD	91	Female	CAD/PAD	—	—
7	DEL	82	Male	PAD	Chronic myelomonocytic leukemia	—
8	UPD/AMP	72	Male	CAD/CAR/PAD	Chronic anemia (?iron/vitamin B_12_ deficiency)	—
8	UPD	81	Male	CAR/PAD	Mantle cell lymphoma	Prostate cancer
11	UPD	65	Female	CAD/CAR/PAD	—	Non–small cell lung carcinoma and ulcerative colitis
14	DEL	85	Female	PAD/stroke	Polycythemia vera	—
14	UPD	71	Male	None	—	—
15^a^	UPD	84	Male	CAD/PAD	Myelodysplastic/myeloproliferative disorder, unclassified	Prostate cancer
15	UPD	85	Female	None	—	Atrial fibrillation and pulmonary arterial hypertension secondary to COPD
15	UPD	73	Male	CAD/PAD	—	—
18	UPD	73	Male	CAD/CAR/PAD	—	Non–small cell lung and prostate cancer
19	UPD	88	Male	CAD/PAD	Thrombocytopenia	Amyloid cardiomyopathy
20	DEL	70	Female	None	Polycythemia vera	Pulmonary arterial hypertension, multiple sclerosis, and pyoderma gangrenosum
20	DEL	75	Male	PAD	Polycythemia vera	Prostate cancer
20	DEL	87	Male	CAD/PAD	Essential thrombocythemia	Prostate cancer
20^a^	DEL	84	Male	CAD/PAD	Myelodysplastic/myeloproliferative disorder, unclassified	Prostate cancer
20	DEL	72	Female	PAD	None (persistently elevated ESR of unknown etiology)	Premature atherosclerosis: PAD diagnosed at age 62
20	UPD	70	Male	CAD/PAD	Polycythemia of unclear etiology (?COPD/sleep apnea)	Abdominal aortic aneurysm
21	UPD	55	Male	—	—	Acoustic neuroma
22	UPD	53	Female	PAD	—	Premature atherosclerosis—PAD diagnosed at age 52
22	UPD	53	Female	PAD	—	Premature atherosclerosis—PAD diagnosed at age 47, severe cognitive disorder (cerebral small vessel disease), and myotonia congenita
22	UPD	90	Male	PAD/stroke	Chronic hemolytic anemia (cold agglutinin antibodies)	Rheumatoid arthritis
22	UPD	72	Male	CAD/PAD	Polycythemia vera	Thromboembolic pulmonary arterial hypertension

Abbreviations: AMP, amplification; CAD, coronary artery disease; CAR, carotid artery stenosis; COPD, chronic obstructive pulmonary disease; DEL, mosaic deletion; PAD, peripheral arterial disease; UPD, unipaternal disomy including both mosaic UPD and unipaternal isodisomy.

a One patient had both UPD of Ch15 and DEL of Ch20.

Of the 25 patients with autosomal chromosomal abnormalities, 10 patients (40%) had a hematologic and/or a solid organ malignancy. Four patients (16%) had a hematologic malignancy only as follows: Ch7 DEL, chronic myelomonocytic leukemia; Ch14 DEL, polycythemia vera; Ch20 DEL, polycythemia vera; and Ch22 UPD, polycythemia vera. Solid organ malignancies were noted in 2 patients (8%): Ch11 UPD, non–small cell lung carcinoma; mosaic Ch18 UPD, non–small cell lung carcinoma/prostate cancer. Both hematologic and solid organ malignancies were observed in 4 patients (16%) as follows: Ch8 UPD/AMP, mantle cell lymphoma/ prostate cancer; Ch20 DEL, polycythemia vera/prostate cancer; Ch20 DEL, essential thrombocythemia/prostate cancer; Ch20 DEL and Ch15 UPD, myeloproliferative/myelodysplastic syndrome, unclassified/prostate cancer.

Of the 50 age-, sex-, and PAD case-matched participants without chromosomal abnormalities, a hematologic malignancy was present in only 1 patient, a significantly lower prevalence of hematologic malignancies than in patients with chromosomal abnormalities (2% vs 32%, respectively, *P* < .001). Although solid organ malignancies were slightly less common in patients without chromosomal abnormalities (n = 10, 20%) compared to patients with chromosomal abnormalities (n = 6, 24%), this difference was not statistically significant (*P* = .69). Solid organ malignancies in the 50 age-, sex-, and PAD case-matched participants without chromosomal abnormalities included prostate cancer (n = 7), colon cancer (n = 1), renal cell carcinoma (n = 1), and endometrial adenocarcinoma (n = 1).

**Figure 2.**
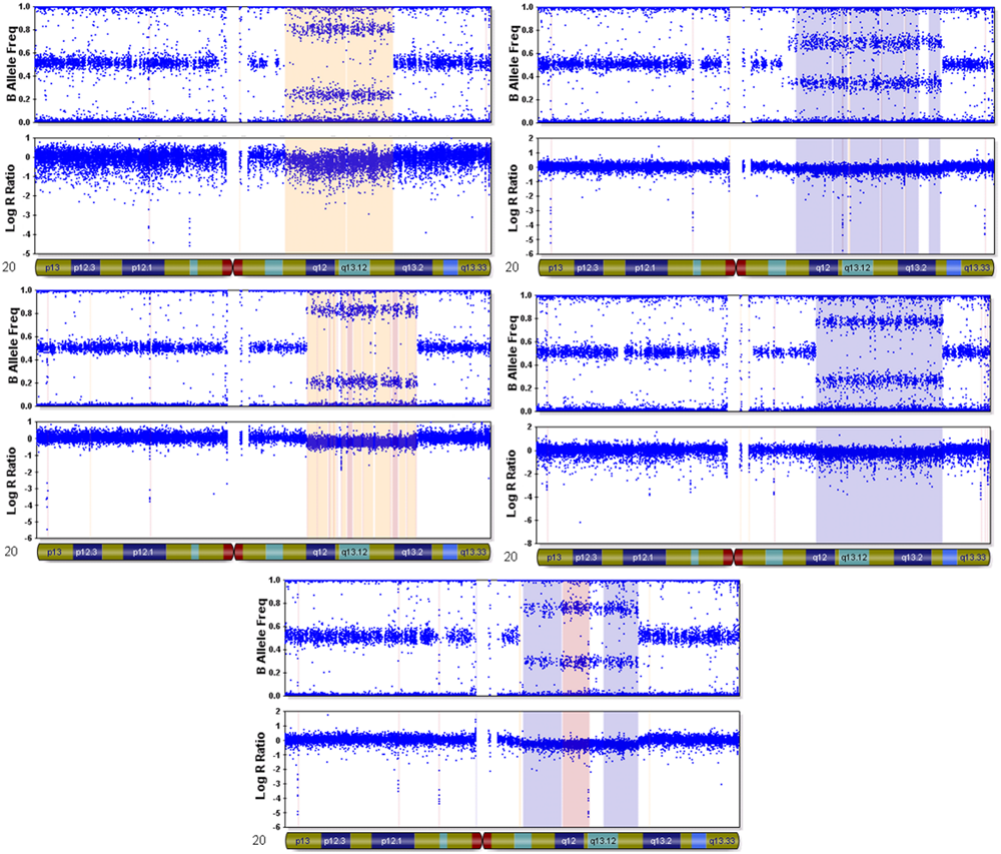
Intensity plots of the detected mosaic chromosome 20q deletions. Note the abrupt decrease in LRR in all plots with an accompanying intermediate BAF indicative of mosaic status.

Of 5 patients (3 men and 2 women) with overlapping mosaic deletions of Ch20q, 4 had a myeloproliferative disease (MPD) as mentioned above (polycythemia vera [n = 2], essential thrombocythemia [n = 1], and myeloproliferative/myelodysplastic syndrome, unclassified [n = 1]). All men in this group had a history of prostate cancer in addition to MPD. One of the women in this group with MPD had history of prostate cancer in her father. The only patient without MPD had a persistently elevated erythrocyte sedimentation rate of unknown etiology and severe PAD. Figure 2 displays the observed deletions in patients with Ch20q deletions as evaluated by intensity plot analyses. The range of Ch20q deletions varied but a common deleted region encompassing approximately 10 Mb (38511024 bp → 48638502 bp) was identified and included 192 genes. An illustration of the common deleted region and the involved genes and their Ensembl identification numbers are provided in the supplementary material.

Of 18 patients with UPD, 14 had atherosclerotic vascular diseases (coronary artery disease, PAD, or stroke). Three of the patients with UPD had a hematologic malignancy whereas 4 had a solid organ malignancy. Two women with UPD of Ch22q had severe premature atherosclerosis necessitating revascularization for PAD at ages 47 and 52. One patient with deletion of Ch7 had chronic myelomonocytic leukemia and cytogenetic analysis (performed as a part of clinical evaluation) had confirmed Ch7 monosomy. Table 1 summarizes the major clinical diagnoses for all patients with chromosomal abnormalities in our GWAS.

## Discussion

Given the proliferation of high-density genome-wide genotyping studies, an increasing number of mosaic autosomal chromosomal abnormalities are being detected. However, the clinical correlates of these abnormalities in study participants are not yet fully characterized. In the present study, we leveraged an EMR-linked GWAS of 3336 adults to identify the major clinical conditions associated with these abnormalities. We found that 0.75% of the study participants (n = 25) had autosomal chromosomal abnormalities. Linkage to the EMR enabled ascertainment of clinical features of these 25 patients beyond PAD case–control status. Compared to 50 age-, sex-, and case-matched participants without chromosomal abnormalities, hematologic malignancies were more frequent among patients with chromosomal abnormalities (32% vs 2%, *P* < .001). Solid organ malignancies were slightly more frequent in patients with chromosomal abnormalities but the difference was not statistically significant (24% vs 20%, *P* = .69).

In 2 large studies^10,12^ assessing chromosomal abnormalities in GWAS, such abnormalities were present in 0.80% to 0.89% of participants. The first of these^12^ evaluated 31 717 cancer cases and 26 136 controls and found 517 patients (0.89%) had at least 1 chromosomal abnormality. Among patients with hematologic malignancies, nearly 20% had a chromosomal abnormality compared to 0.76% in cancer-free controls. These abnormalities were also associated with an increased rate of solid organ malignancies compared to cancer-free patients. Older age was associated with increased incidence of these abnormalities (0.23% for patients <50 years compared to 1.91% for patients 75-79 years old).^12^ The second study^10^ included more than 50 000 participants and demonstrated a 10-fold higher odds for a hematologic malignancy among patients with mosaic chromosomal abnormalities versus nonmosaic individuals. These abnormalities increased in prevalence with increasing age and were also associated with hematologic and solid organ malignancies. Our results are similar with regard to the higher prevalence of hematologic malignancies although the frequency of solid organ malignancies was not significantly different, likely due to the smaller sample size in our study.

A potential novel finding of our study is a possible association between Ch20q deletion, MPD, and prostate cancer. A myeloproliferative disorder was present in 4 (of 5) of these patients and prostate cancer in all 3 male patients. Interstitial deletions of Ch20q have been previously described in MPD and to a lesser extent in myelodysplastic syndromes.^13^ Although Ch20q deletions are not pathognomonic for polycythemia vera (PV), up to 10% of PV patients have Ch20q deletions compared to 5% in patients with myelodysplastic syndrome.^14^ Among patients with myelofibrosis (primary or secondary to PV and essential thrombocythemia), Ch20q deletion was the most frequent cytogenetic abnormality, occurring in up to 36% of affected patients.^15^ Ch20q deletions have also been reported to be associated with acute lymphocytic and lymphoblastic leukemias.^16^

The tumorigenic pathways underlying Ch20q deletions are still being investigated. Ch20q deletions may lead to loss of tumor suppressor genes and thereby increase the risk for developing hematologic malignancies.^17,18^ The common deleted region in MPD patients was evaluated in several previous studies and a variety of break points in the region were noted with no homozygous deletion in Ch20q characterized till date.^10,19^ Another group of researchers^20^ evaluated the common retained regions of Ch20q in addition to the previously described common deleted region. They hypothesized that these common retained regions may contain oncogenes that may be overexpressed following Ch20q deletion and thereby contribute to the development of MPD. Other studies suggested that Ch20q deletion and subsequent genomic rearrangement may affect retained neighboring genes and result in either inhibition of tumor suppressor genes (such as *DIDO1*) or overexpression of other retained candidate oncogenes.^20-22^

Several linkage studies demonstrated that 20q13 locus was associated with prostate cancer.^23-26^ Ch20q gains have also been demonstrated to be associated with several malignancies including prostate cancer.^27-30^ However, Ch20q deletions were less frequently associated with prostate cancer compared to Ch20q gains.^31,32^ Considering that MPD and prostate cancer were associated with Ch20q deletions in our patients, altered gene expression of the retained regions of Ch20q is a more likely explanation for the observed phenotype. Ch20q deletion seems to result in either inhibition of tumor suppressor gene(s) or overexpression of oncogene(s) of the common retained regions of Ch20q. Identification of genes and pathways that might potentially lead to prostate cancer and MPD in the setting of Ch20q deletion requires further investigation.

## Psychosocial and Ethical Implications

The incidental finding of autosomal chromosomal abnormalities in a GWAS has significant psychosocial and ethical implications. For example, one of the patients with Ch20q deletions did not have a diagnosis of malignancy in EMR but was noted to have persistently elevated erythrocyte sedimentation rate ranging between 35 and 50 mm/1 hour as well as a marginally elevated neutrophil count (5.6 × 10^9^/L to 8.3 × 10^9^/L), raising the concern that the patient could have an underlying malignancy or was at potential risk of developing malignancy in the future. At present, there are no prospective data that provide the relative risk for future hematologic or solid organ malignancies in otherwise asymptomatic patients with chromosomal abnormalities. There is also no consensus on how to appropriately manage such incidental findings nor any standardized protocols for synthesizing, analyzing, and disclosing this genetic information in the clinical setting.^33,34^ Furthermore, whether the reportable information would ultimately be “actionable” or linked to downstream screening, diagnostic studies, or treatments remains uncertain. In the example described above, the incidental finding was not reported due to the paucity of correlative clinical data and the lack of evidence supporting downstream implications of Ch20q deletions as well as other genetic abnormalities. Additionally, informed consent (including for this study) often does not specify returning results to study participants.^35^ Where disclosure is required, the use of laboratory tests that have been certified by Clinical Laboratory Improvement Act/Amendment is recommended to ensure federal regulations and proficiency standards are upheld.

We expect the number of incidentally found chromosomal abnormalities to increase as more GWAS are carried out. Prospective follow-up of patients with such chromosomal abnormalities will be required to ascertain outcomes including the risk of developing malignancy. Only then will we be able to understand how these abnormalities affect otherwise asymptomatic patients and whether further medical intervention is justified in these patients or not. Until a more robust understanding of the clinical relevance of genetic variants is obtained, the implications of these discoveries will remain unclear as would the investigator’s obligation to disclose those findings to study subjects. Nonetheless, research study participants’ demand for insight into these findings will likely increase as will the demand for disclosure.^36^

## Conclusion

In a GWAS of 3336 adults, 0.75% (n = 25) had autosomal chromosomal abnormalities, and of these 40% had a hematologic and/or a solid organ malignancy. Chromosomal abnormalities were significantly associated with the presence of hematologic malignancies. We also noted a possible association of Ch20q deletions with MPD and prostate cancer highlighting the potential of EMR-linked GWAS to uncover new genotype–phenotype correlations. Further research using larger cohorts will be required to confirm this finding. As the number of incidentally found chromosomal abnormalities in otherwise asymptomatic patients is expected to increase, there is a pressing need for prospective studies that evaluate the outcomes and downstream implications of such abnormalities.

## References

[1] Manolio TA Genomewide association studies and assessment of the risk of disease. N Engl J Med. 2010;363:166-176. 20647212 10.1056/NEJMra0905980

[2] Ding K Kullo IJ Genome-wide association studies for atherosclerotic vascular disease and its risk factors. Circ Cardiovasc Genet. 2009;2:63-72. 19750184 10.1161/CIRCGENETICS.108.816751 PMC2740629

[3] Rodriguez-Santiago B Malats N Rothman N Mosaic uniparental disomies and aneuploidies as large structural variants of the human genome. Am J Hum Genet. 2010;87:129-138. 20598279 10.1016/j.ajhg.2010.06.002 PMC2896781

[4] Kho AN Pacheco JA Peissig PL Electronic medical records for genetic research: results of the eMERGE consortium. Sci Transl Med. 2011;3:79re1. 10.1126/scitranslmed.3001807 PMC3690272 21508311

[5] McCarty CA Chisholm RL Chute CG The eMERGE Network: a consortium of biorepositories linked to electronic medical records data for conducting genomic studies. BMC Med Genomics. 2011;4:13. 21269473 10.1186/1755-8794-4-13 PMC3038887

[6] Kullo IJ Fan J Pathak J Savova GK Ali Z Chute CG Leveraging informatics for genetic studies: use of the electronic medical record to enable a genome-wide association study of peripheral arterial disease. J Am Med Inform Assoc. 2010;17:568-574. 20819866 10.1136/jamia.2010.004366 PMC2995686

[7] Ding K Shameer K Jouni H Genetic loci implicated in erythroid differentiation and cell cycle regulation are associated with red blood cell traits. Mayo Clin Proc. 2012;87:461-474. 22560525 10.1016/j.mayocp.2012.01.016 PMC3538470

[8] Crosslin DR McDavid A Weston N Genetic variants associated with the white blood cell count in 13,923 subjects in the eMERGE Network. Hum Genet. 2012;131:639-652. 22037903 10.1007/s00439-011-1103-9 PMC3640990

[9] Denny JC Ritchie MD Crawford DC Identification of genomic predictors of atrioventricular conduction: using electronic medical records as a tool for genome science. Circulation. 2010;122:2016-2021. 21041692 10.1161/CIRCULATIONAHA.110.948828 PMC2991609

[10] Laurie CC Laurie CA Rice K Detectable clonal mosaicism from birth to old age and its relationship to cancer. Nat Genet. 2012;44:642-650. 22561516 10.1038/ng.2271 PMC3366033

[11] Durinck S Moreau Y Kasprzyk A BioMart and Bioconductor: a powerful link between biological databases and microarray data analysis. Bioinformatics. 2005;21:3439-3440. 16082012 10.1093/bioinformatics/bti525

[12] Jacobs KB Yeager M Zhou W Detectable clonal mosaicism and its relationship to aging and cancer. Nat Genet. 2012;44:651-658. 22561519 10.1038/ng.2270 PMC3372921

[13] An Q Wright SL Moorman AV Heterogeneous breakpoints in patients with acute lymphoblastic leukemia and the dic(9;20)(p11-13;q11) show recurrent involvement of genes at 20q11.21. Haematologica. 2009;94:1164-1169. 19586940 10.3324/haematol.2008.002808 PMC2719040

[14] Asimakopoulos FA White NJ Nacheva E Green AR Molecular analysis of chromosome 20q deletions associated with myeloproliferative disorders and myelodysplastic syndromes. Blood. 1994;84:3086-3094. 7949181

[15] Hussein K Van Dyke DL Tefferi A Conventional cytogenetics in myelofibrosis: literature review and discussion. Eur J Haematol. 2009;82:329-338. 19141119 10.1111/j.1600-0609.2009.01224.x

[16] Mitelman F Mertens F Johansson B A breakpoint map of recurrent chromosomal rearrangements in human neoplasia. Nat Genet. 1997;15 Spec No:417-74. 9140409 10.1038/ng0497supp-417

[17] Bench AJ Aldred MA Humphray SJ A detailed physical and transcriptional map of the region of chromosome 20 that is deleted in myeloproliferative disorders and refinement of the common deleted region. Genomics. 1998;49:351-362. 9615219 10.1006/geno.1998.5231

[18] Bench AJ Li J Huntly BJ Characterization of the imprinted polycomb gene L3MBTL, a candidate 20q tumour suppressor gene, in patients with myeloid malignancies. Br J Haematol. 2004;127:509-518. 15566354 10.1111/j.1365-2141.2004.05278.x

[19] Bench AJ Nacheva EP Hood TL Chromosome 20 deletions in myeloid malignancies: reduction of the common deleted region, generation of a PAC/BAC contig and identification of candidate genes. UK Cancer Cytogenetics Group (UKCCG). Oncogene. 2000;19:3902-3913. 10952764 10.1038/sj.onc.1203728

[20] Douet-Guilbert N Basinko A Morel F Chromosome 20 deletions in myelodysplastic syndromes and Philadelphia-chromosome-negative myeloproliferative disorders: characterization by molecular cytogenetics of commonly deleted and retained regions. Ann Hematol. 2008;87:537-544. 18350294 10.1007/s00277-008-0462-3

[21] Futterer A Campanero MR Leonardo E Dido gene expression alterations are implicated in the induction of hematological myeloid neoplasms. J Clin Invest. 2005;115:2351-2362. 16127461 10.1172/JCI24177 PMC1190370

[22] Tefferi A Novel mutations and their functional and clinical relevance in myeloproliferative neoplasms: JAK2, MPL, TET2, ASXL1, CBL, IDH and IKZF1. Leukemia. 2010;24:1128-1138. 20428194 10.1038/leu.2010.69 PMC3035972

[23] Berry R Schroeder JJ French AJ Evidence for a prostate cancer-susceptibility locus on chromosome 20. Am J Hum Genet. 2000;67:82-91. 10820130 10.1086/302994 PMC1287105

[24] Brown WM Lange EM Chen H Hereditary prostate cancer in African American families: linkage analysis using markers that map to five candidate susceptibility loci. Br J Cancer. 2004;90:510-514. 14735201 10.1038/sj.bjc.6601417 PMC2410149

[25] Cunningham JM McDonnell SK Marks A Genome linkage screen for prostate cancer susceptibility loci: results from the Mayo Clinic Familial Prostate Cancer Study. Prostate. 2003;57:335-346. 14601030 10.1002/pros.10308

[26] Zheng SL Xu J Isaacs SD Evidence for a prostate cancer linkage to chromosome 20 in 159 hereditary prostate cancer families. Hum Genet. 2001;108:430-435. 11409871 10.1007/s004390100513

[27] Hodgson JG Chin K Collins C Gray JW Genome amplification of chromosome 20 in breast cancer. Breast Cancer Res Treat. 2003;78:337-345. 12755492 10.1023/a:1023085825042

[28] Rae FK Hooper JD Nicol DL Clements JA Characterization of a novel gene, STAG1/PMEPA1, upregulated in renal cell carcinoma and other solid tumors. Mol Carcinog. 2001;32:44-53. 11568975 10.1002/mc.1063

[29] Uchida M Tsukamoto Y Uchida T Genomic profiling of gastric carcinoma in situ and adenomas by array-based comparative genomic hybridization. J Pathol. 2010;221:96-105. 20217874 10.1002/path.2686

[30] Xu LL Shanmugam N Segawa T A novel androgen-regulated gene, PMEPA1, located on chromosome 20q13 exhibits high level expression in prostate. Genomics. 2000;66:257-263. 10873380 10.1006/geno.2000.6214

[31] Brookman-Amissah N Duchesnes C Williamson MP Genome-wide screening for genetic changes in a matched pair of benign and prostate cancer cell lines using array CGH. Prostate Cancer Prostatic Dis. 2005;8:335-343. 16130014 10.1038/sj.pcan.4500826

[32] Cher ML MacGrogan D Bookstein R Brown JA Jenkins RB Jensen RH Comparative genomic hybridization, allelic imbalance, and fluorescence in situ hybridization on chromosome 8 in prostate cancer. Genes Chromosomes Cancer. 1994;11:153-162. 7530484 10.1002/gcc.2870110304

[33] Kullo IJ Jarvik GP Manolio TA Williams MS Roden DM Leveraging the electronic health record to implement genomic medicine. Genet Med. 2013;15:270-271. 23018749 10.1038/gim.2012.131 PMC4206937

[34] Wolf SM Crock BN Van Ness B Managing incidental findings and research results in genomic research involving biobanks and archived data sets. Genet Med. 2012;14:361-384. 22436882 10.1038/gim.2012.23 PMC3597341

[35] Fullerton SM Wolf WA Brothers KB Return of individual research results from genome-wide association studies: experience of the Electronic Medical Records and Genomics (eMERGE) Network. Genet Med. 2012;14:424-431. 22361898 10.1038/gim.2012.15 PMC3723451

[36] Bollinger JM Scott J Dvoskin R Kaufman D Public preferences regarding the return of individual genetic research results: findings from a qualitative focus group study. Genet Med. 2012;14:451-457. 22402755 10.1038/gim.2011.66 PMC3927946

